# Identification of prior dengue-naïve Dengvaxia recipients with an increased risk for symptomatic dengue during fever surveillance in the Philippines

**DOI:** 10.3389/fimmu.2023.1202055

**Published:** 2023-07-24

**Authors:** Yu-Ching Dai, Ava Kristy Sy, Mario Jiz, Jih-Jin Tsai, Joan Bato, Mary Ann Quinoñes, Mary Anne Joy Reyes, Wei-Kung Wang

**Affiliations:** ^1^ Department of Tropical Medicine, Medical Microbiology and Pharmacology, John A. Burns School of Medicine, University of Hawaii at Manoa, Honolulu, HI, United States; ^2^ National Reference Laboratory for Dengue and Other Arboviruses, Virology Department, Research Institute for Tropical Medicine, Muntinlupa, Philippines; ^3^ Immunology Department, Research Institute for Tropical Medicine, Muntinlupa, Philippines; ^4^ Tropical Medicine Center, Kaohsiung Medical University Hospital, Kaohsiung, Taiwan; ^5^ Division of Infectious Diseases, Department of Internal Medicine, Kaohsiung Medical University Hospital, Kaohsiung, Taiwan; ^6^ School of Medicine, College of Medicine, Kaohsiung Medical University, Kaohsiung, Taiwan

**Keywords:** dengue virus, vaccine, Dengvaxia, serostatus, enzyme-linked immunosorbent assay

## Abstract

**Introduction:**

Dengue virus (DENV) is the leading cause of mosquito-borne viral diseases in humans. Dengvaxia, the first licensed dengue vaccine, is recommended for DENV-seropositive individuals aged 9–45 years. In the Philippines, Dengvaxia was administered to more than 830,000 children without prior serological testing in 2016–2017. Subsequently, it was revealed that DENV-seronegative children who received Dengvaxia developed severe disease following breakthrough DENV infection. As a result, thousands of children participating in the mass vaccination campaign were at higher risk of severe dengue disease. It is vital that an assay that identifies baseline DENV-naïve Dengvaxia recipients be developed and validated. This would permit more frequent and extensive assessments and timely treatment of breakthrough DENV infections.

**Methods:**

We evaluated the performance of a candidate assay, the DENV1–4 nonstructural protein 1 (NS1) IgG enzyme-linked immunosorbent assay (ELISA), developed by the University of Hawaii (UH), using well-documented serum/plasma samples including those >20 years post-DENV infection, and tested samples from 199 study participants including 100 Dengvaxia recipients from the fever surveillance programs in the Philippines.

**Results:**

The sensitivity and specificity of the assay were 96.6% and 99.4%, respectively, which are higher than those reported for pre-vaccination screening. A significantly higher rate of symptomatic breakthrough DENV infection was found among children that were DENV-naïve (10/23) than among those that were DENV-immune (7/53) when vaccinated with Dengvaxia (p=0.004, Fisher’s exact test), demonstrating the feasibility of the assay and algorithms in clinical practice.

**Conclusion:**

The UH DENV1–4 NS1 IgG ELISA can determine baseline DENV serostatus among Dengvaxia recipients not only during non-acute dengue but also during breakthrough DENV infection, and has implications for assessing the long-term safety and effectiveness of Dengvaxia in the post-licensure period.

## Introduction

The four serotypes of dengue virus (DENV; DENV1–DENV4) cause an estimated 390 million infections worldwide each year (1, 2). Although most DENV infections are not apparent, approximately 25% result in clinical disease, ranging from a self-limited illness, known as dengue, to more severe and potentially life-threatening disease, known as dengue with warning signs and severe dengue, respectively (1–3).

After a primary DENV (pDENV) infection, individuals develop long-term protection against the infecting serotype. During secondary DENV (sDENV) infection with a different serotype, individuals are at higher risk of developing severe disease than those experiencing pDENV infection (1). No licensed antiviral drugs are currently available for dengue treatment. Although several dengue vaccine candidates have completed different phases of clinical trials, Dengvaxia, a chimeric yellow fever-tetravalent dengue vaccine, was the first dengue vaccine licensed in 20 countries (4, 5). Based on initial reports, Dengvaxia was recommended for individuals aged 9–45 years in 2016 (4–7).

In the Philippines, a Dengvaxia school-based vaccination program was launched in April 2016 among 9–10-year-old children with >830,000 children receiving at least one dose (~420,000 one dose, ~49,000 two doses, and ~370,000 three doses) (5, 8–10). After this program was initiated, a DENV nonstructural protein 1 (NS1) IgG enzyme-linked immunosorbent assay (ELISA) was employed in a *post-hoc* case-control study to determine the baseline DENV serostatus in samples collected 13 months after the first dose of Dengvaxia. The results showed that DENV-seronegative children receiving Dengvaxia were at higher risk of hospitalization and severe dengue during breakthrough DENV infections (5, 11, 12), resulting in the cancellation of the Dengvaxia vaccination program and an overall increase in vaccine hesitancy in the Philippines.

Subsequently, the recommendation for Dengvaxia was revised by the World Health Organization and administration of the vaccine was limited to DENV-seropositive individuals aged 9–45 years (5, 8, 9, 13). A pre-vaccination screening strategy using assays with high specificity (≥98.0%) to avoid erroneous vaccination of individuals without prior DENV infection and high sensitivity (≥95.0%) to detect individuals with a single prior DENV infection has been proposed (14, 15). Several serological tests have been reported to determine the DENV serostatus for pre-vaccination screening, including rapid diagnostic tests (RDTs) and ELISAs (16–21).

Recent meta-analyses have identified sDENV infection as a prognostic marker for severe dengue and recommended the inclusion of sDENV infection in the bedside scoring system to facilitate triage and timely treatment of patients with dengue prior to progression to severe dengue (22–24). As such, a serological test that can determine DENV serostatus prior to receiving Dengvaxia is critically needed; such a test should have high sensitivity and specificity, allowing for the identification of baseline DENV-naïve Dengvaxia recipients that would experience an sDENV infection during breakthrough infection, consequently being at high risk of severe dengue (8, 25). A previously reported DENV NS1 IgG ELISA was used to test 13-month samples collected during the Dengvaxia vaccine trials (10, 11). At present, it is unclear whether this test can be applied to Dengvaxia recipients in the real world, where some received only one or two doses >13 months after vaccination, if it can be applied to individuals that present with breakthrough DENV infection, or if it can help assess the long-term safety and effectiveness of Dengvaxia (26, 27).

Within the genus *Flavivirus* of the family *Flaviviridae*, there are several mosquito- or tick-borne viruses that cause prominent human diseases, including the four DENV serotypes, Zika virus (ZIKV), West Nile virus (WNV), Japanese encephalitis virus (JEV), yellow fever virus (YFV), and tick-borne encephalitis virus (28). As the major target of the antibody response following DENV infection, the envelope (E) protein has been employed as the main antigen for serological tests, including the use of recombinant E protein, inactivated virions, or virus-like particles (VLPs) (28). Due to the cross-reactivity of anti-E antibodies to various flaviviruses, different or modified antigens, such as NS1 protein, fusion-loop (FL)-mutated recombinant E proteins, and VLPs, have been developed (29–31). We previously reported that ELISAs based on DENV1–4 NS1 protein and DENV1 FL-mutated VLPs could detect DENV infection with a sensitivity and specificity of 95.6%/89.5% and 100.0%/93.3%, respectively (32, 33).

The objectives of this study were to 1) evaluate the performance of our NS1 IgG ELISA (developed by the University of Hawaii; UH), designated as UH DENV1–4 NS1 IgG ELISA, by using well-documented samples of different flavivirus infections with known sampling times; 2) investigate whether it can be employed to determine the baseline DENV serostatus of Dengvaxia recipients in real-world settings, some of whom received only one or two doses >13 months after the first dose; and 3) determine the baseline DENV serostatus during both non-acute dengue and acute dengue situations. As Dengvaxia presents the premembrane/E proteins of DENV and nonstructural proteins including NS1 of YFV, we hypothesized that for Dengvaxia recipients without acute dengue, failure to detect DENV NS1 IgG antibodies would indicate a child vaccinated when DENV-naïve, whereas detection of DENV NS1 IgG would indicate a child vaccinated with prior DENV infection(s). For Dengvaxia recipients bled during an acute DENV infection, detection of DENV NS1 IgG could be due to a previous DENV infection or induced by a current DENV infection depending on the sampling day.

## Materials and methods

### Samples from participants

The study of coded serum or plasma samples was approved by the Institutional Review Boards of the UH (2022-00201, 2021-00947, CHS #17568), Research Institute for Tropical Medicine (RITM), Philippines (2019-042), and Kaohsiung Medical University Hospital (KMUH; KMUH-IRB-960195 and KMUH-IRB-[I]-20170185). The numbers, sources, sampling times, and confirmation methods for the different panels of control samples are summarized in **Supplementary Table 1**. Samples from a DENV seroprevalence study in Kaohsiung, Taiwan, were confirmed by a microneutralization test (to DENV) as pDENV, sDENV, or DENV-negative (34, 35), and the sampling time was available based on questionnaires from study participants. Samples of reverse transcription-polymerase chain reaction (RT-PCR)-confirmed DENV cases were obtained from Taiwan, Hawaii, and Nicaragua prior to the Zika outbreak in 2015−2016 (32, 34, 36), and JEV cases were obtained from the KMUH, Taiwan. Samples from a ZIKV study in Salvador, Brazil, were confirmed by a microneutralization test (to ZIKV and DENV) as primary ZIKV (pZIKV) and ZIKV with previous DENV (ZIKVwprDENV) infection (37). Samples from blood donors that tested positive for WNV transcription-mediated amplification and IgM and IgG antibodies were designated as primary WNV (pWNV) infection (32). Samples from YF-17D vaccine recipients (n=19) were tested using YFV NS1 IgG ELISA but were not included as a control panel because of the unknown history of other flavivirus infections.

### Fever surveillance in the Philippines

In 2018, the Department of Health in the Philippines initiated a fever surveillance program for Dengvaxia recipients. The inclusion criteria were Dengvaxia recipients with acute febrile illness. Administration of Dengvaxia was verified using vaccine cards or a list from the national vaccination program (Epidemiology Bureau, the Philippines). The exclusion criteria were patients that did not receive Dengvaxia. After Dengvaxia recipients presented with symptoms compatible with dengue at any health facility in the Philippines (3), blood samples were collected and sent to RITM, the national reference laboratory, for diagnostic testing. Participants testing positive, using either DENV RT-PCR or Panbio dengue IgM-capture ELISA (Abbott, South Korea), were defined as laboratory-confirmed acute dengue cases, and participants testing negative using both tests were classified as non-acute dengue or other febrile illnesses (38, 39). The day of symptom (fever) onset was designated as day 1. A total of 199 participants (100 Dengvaxia and 99 non-Dengvaxia recipients) were included in this study (**Table 1**). Samples from Dengvaxia recipients, including 59 non-acute dengue and 41 acute dengue cases, were selected by simple random sampling from >3,000 samples of Dengvaxia recipients received at the RITM between January 2018 and May 2019. Samples from non-Dengvaxia recipients (all age groups) including 50 non-acute dengue and 49 acute dengue cases (49 first-time point and 20 second-time point samples) were randomly selected from the existing dengue surveillance program at the RITM. The non-Dengvaxia group served as a comparative group to assess the DENV seroprevalence rate in this population.

**Table 1:** Samples from fever surveillance programs in the Philippines.

Group^a^	Subgroup	Laboratory tests^b^	No. of subjects/samples	Sampling day^c^ (post-symptom onset)
Non-Dengvaxia recipients	Non-acute dengue	DENV RT-PCR (**−**) and DENV IgM (**−**)	50/50	day 2**−**14
	Acute dengue (first sample)	DENV RT-PCR (+) or DENV IgM (+)	49/49	day 3**−**10 acute to early convalescent-phase
	Acute dengue (second sample)	DENV RT-PCR (+) or DENV IgM (+)	20/20	day 6**−**18 acute to convalescent-phase
Dengvaxia recipients	Non-acute dengue	DENV RT-PCR (**−**) and DENV IgM (**−**)	59/59	day 7**−**32
	Acute dengue (first sample)	DENV RT-PCR (+) or DENV IgM (+)	41/41	day 7**−**21 acute to convalescent-phase

a Dengvaxia and non-Dengvaxia recipients were from the fever surveillance program for Dengvaxia and dengue surveillance program, respectively, in the Philippines.

b DENV RT-PCR test and Panbio dengue IgM-capture ELISA were performed at the RITM. Either one tested positive was designated as acute dengue and both tested negative as non-acute dengue.

c The day of symptom (fever) onset was designated as day 1.

### NS1 IgG ELISAs

DENV1−DENV4 and YFV NS1 proteins were purchased from Native Antigen (Oxford, UK). To increase the possibility of identifying previous DENV infections, we increased the amount of NS1 proteins coated on 96-well plates (24/12/24/12 ng of pooled DENV1/2/3/4 NS1 proteins per well, respectively, or 50 ng of YFV NS1 protein per well) compared to our previously reported NS1 IgG ELISA (32). This was followed by incubation with a blocking buffer, primary (serum or plasma at 1:400) and secondary (anti-human IgG conjugated with horseradish peroxidase) antibodies, and substrate and stop solution (32). The optical density (OD) at 450 nm was recorded using 630 nm as the reference wavelength (32). Each ELISA plate included two positive controls (confirmed-dengue samples), four negative controls (DENV-negative samples), and test samples; all samples were run in duplicate wells. The OD values were divided by the mean OD value of one positive control (expected OD close to 1), which was run in duplicate on the same plate, to calculate the relative OD (rOD) values for comparison between the plates. The cutoff rOD was defined as the mean rOD value of negatives plus 12 standard deviations, which was, at a minimum, the 99.3rd percentile of the distribution of negative rODs (32, 40). The mean cutoff rOD values of 18 plates were determined as the final cutoff rOD (0.139 and 0.098 for DENV1–4 and YFV NS1 ELISAs, respectively). Each ELISA was performed twice at the UH.

### DENV FL-VLP IgG ELISA

IgG ELISA using DENV1 FL-mutated VLPs was performed as previously described (33). Briefly, DENV1 FL-mutated VLPs (containing W101A and F108A mutations) were coated onto 96-well plates at 4°C overnight, after which the steps previously described were followed (33). The OD read, positive and negative controls, rOD, and cutoff rOD were recorded and calculated as described above, and the mean cutoff rOD from six plates was determined as the final cutoff rOD (0.117).

### Microneutralization test

Microneutralization tests were performed as previously described (35, 37). Briefly, two-fold serial dilutions of serum were mixed with 50 focus-forming units of DENV1 (Hawaii), DENV2 (NGC), DENV3 (CH53489), DENV4 (H241), or ZIKV (PRVABC59) at 37°C for 1 h; the mixtures were added to each well of 96-well plate pre-seeded with Vero cells, followed by incubation at 37°C for 48−70 h, fixation, mouse mAb 4G2 and secondary antibodies (IRDye^®^ 800CW-conjugated goat anti-mouse IgG and DRAQ5™) (35, 37). Signals were detected using a Li Cor Odyssey imager and analyzed using Image Studio (LiCor Bioscience, Lincoln, Nebraska) to determine percent neutralization at different concentrations and 90% neutralization (NT_90_) titers (35, 37).

### Statistical analysis

The odds ratio and 95% confidence intervals (CI) were calculated using Excel. Two-tailed Fisher’s exact test and Mann−Whitney test were used to compare qualitative and quantitative variables, respectively, between the two groups. The Kruskal−Wallis test and chi-square test for trend were used to compare quantitative variables and proportions, respectively, between the three groups (GraphPad Prism 6, GraphPad, Boston, Massachusetts). McNemar’s test was used to compare the sensitivity of two assays within the same group (SPSS 20). Sensitivity and specificity were calculated using different panels of control samples from each individual, and multiple samples from the same individual were counted only once.

## Results

### UH DENV1–4 NS1 IgG ELISA

**Figure 1:**
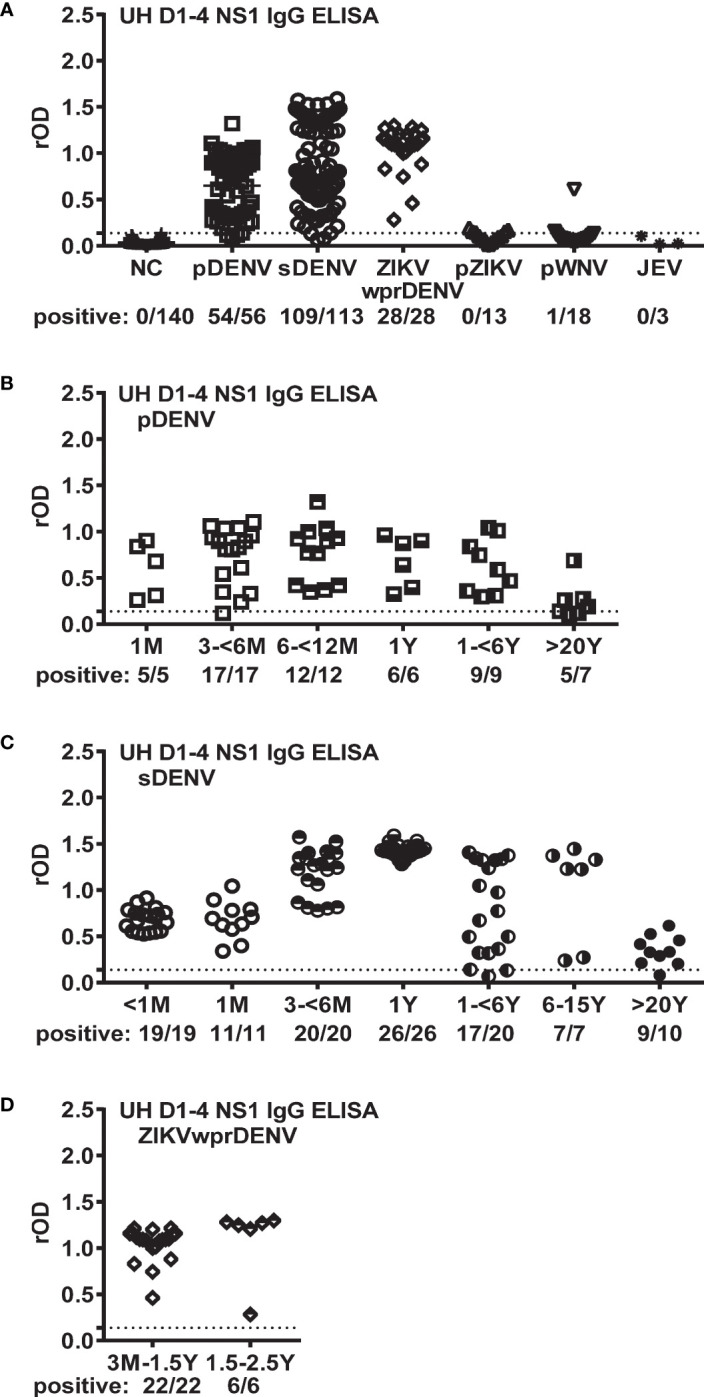
Performance of UH DENV1–4 NS1 IgG ELISA on panels of well-documented serum samples with known flavivirus infections. **(A)** rOD and detection rates in different control panels including DENV (pDENV, sDENV, and ZIKVwprDENV) and non-DENV (DENV-negative, pZIKV, pWNV, and JEV) panels. **(B−D)** rOD and detection rates for samples with different sampling times after symptom onset for the pDENV **(B)**, sDENV **(C)** and ZIKVwprDENV **(D)** panels. Dotted lines indicate the cutoff rOD. Data represent the mean of two experiments (each in duplicate). rOD, relative OD; NC, negative control of DENV-negative samples; pDENV, primary DENV infection; sDENV, secondary DENV infection; pWNV, primary WNV infection; pZIKV, primary ZIKV infection; ZIKVwprDENV, ZIKV infection with previous DENV infection; JEV, JEV infection.

The majority (191/197) of the samples from the DENV panels (pDENV, sDENV, and ZIKVwprDENV) contained anti-DENV NS1 IgG antibodies in one or more tests (**Figure 1A**). Of the 174 sera from the non-DENV panels (DENV-negative, pZIKV, pWNV, and JEV), only 1 contained dengue NS1 IgG antibodies. The overall sensitivity/specificity was 96.6%/99.4% (95% CI=93.9−98.0%/98.3−100.0%). When examining the detection rate over time, we found that the sensitivity declined to 71.4% in samples collected >20 years after symptom onset for the pDENV panel (**Figure 1B**), and the sensitivity was between 85% and 100% from 1–6 years to >20 years for the sDENV panel (**Figure 1C**). Notably, the detection rate remained at 100% up to 2.5 years for the ZIKVwprDENV panel (**Figure 1D**).

### DENV serostatus among non-Dengvaxia recipients

**Figure 2:**
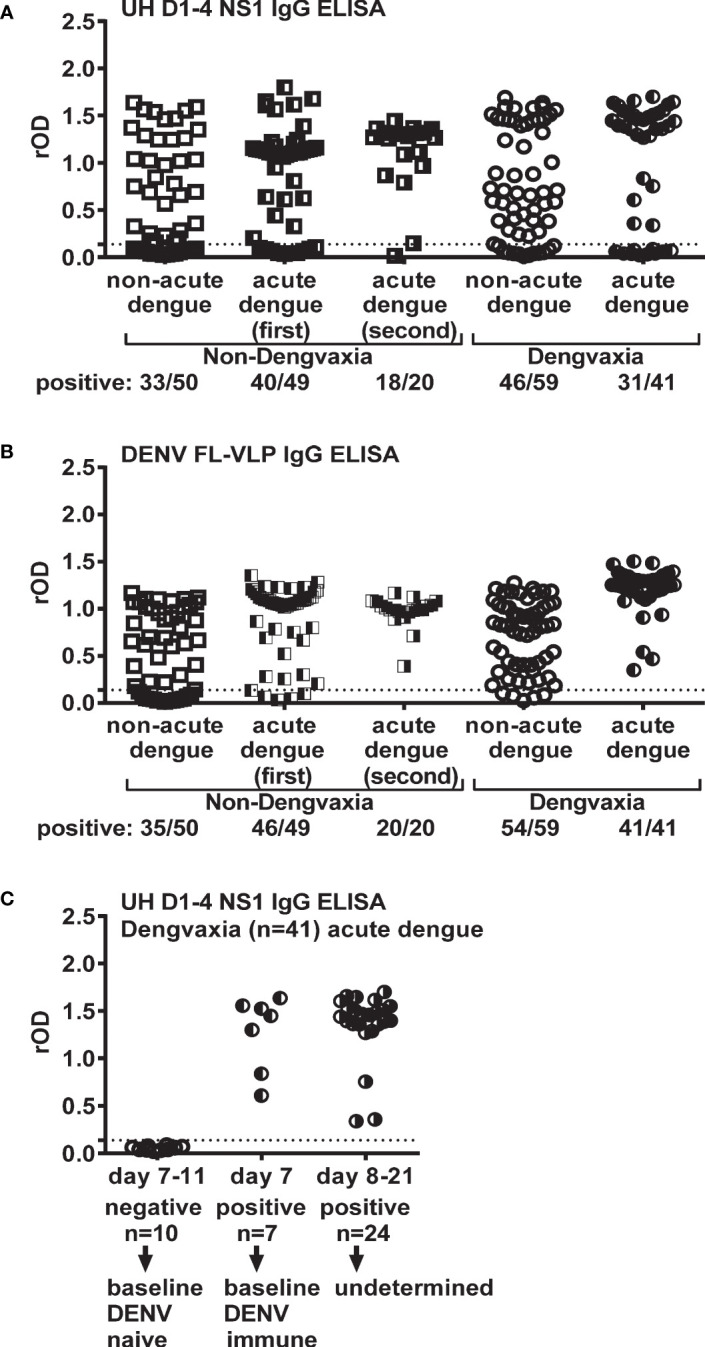
Results of UH DENV1–4 NS1 IgG and DENV FL-VLP IgG ELISAs using samples of participants from the fever surveillance in the Philippines. There are non-Dengvaxia recipients (non-acute dengue and acute dengue first and second time-point subgroups) and Dengvaxia recipients (non-acute dengue and acute dengue subgroups). **(A)** UH DENV1–4 NS1 IgG ELISA, **(B)** DENV FL-VLP IgG ELISA and **(C)** UH DENV1–4 NS1 IgG ELISA for Dengvaxia recipients with acute dengue stratified by different sampling times post-symptom onset. Dotted lines indicate cutoff rOD. Data represent the mean of two experiments (each in duplicate). rOD, relative OD.

Next, we used the UH DENV1–4 NS1 IgG ELISA to examine the DENV serostatus in 99 non-Dengvaxia recipients (**Table 1**). As shown in **Figure 2A**, 33/50 of the non-acute dengue subgroup tested positive, suggesting a DENV seroprevalence rate of 66.0%, which was consistent with the positive rate of 70.0% (35/50) measured using a previously described E protein-based FL-VLP IgG ELISA (**Figure 2B**). As expected, a trend of increasing seropositivity with age was observed (**Supplementary Figure 1**). We next examined the first and second time-point samples from 49 acute dengue cases and found that 81.6% (40/49) of the first and 90.0% (18/20) of the second time-point samples tested positive (**Figure 2A**). A similar trend was observed using FL-VLP IgG ELISA, with positive rates of 93.9% (46/49) and 100.0% (20/20) for the first and second time-point samples, respectively (**Figure 2B**). We did not test for differences or trends, but the results were consistent with the delayed appearance of anti-NS1 IgG during the early convalescent phase or a faster anti-E antibody response (29, 32, 33). This was consistent with the findings of other trials in which DENV RNAemia and NS1 antigenemia were measured. Of note, there was no difference in sensitivity between the UH DENV1–4 NS1 IgG ELISA and FL-VLP IgG ELISA when testing non-Dengvaxia recipients with non-acute dengue (33/50 vs. 35/50) and at the second time point after acute dengue (18/20 vs. 20/20) (p=0.69 and 0.50, respectively, McNemar’s test) except at the first time point after acute dengue (40/49 vs. 46/49) (p=0.03, McNemar’s test).

### DENV serostatus among Dengvaxia recipients

Next, we employed a DENV FL-VLP IgG ELISA to examine whether the anti-E antibody was induced by Dengvaxia. In the non-acute dengue subgroup, 54/59 (91.5%) tested positive, suggesting a seroconversion rate of 91.5%, which can probably be attributed to the fact that most (56.1%) Dengvaxia recipients in this study received only one dose (**Figure 2B**). Of the 57 Dengvaxia recipients, 28/32 (87.5%) of those who received one dose seroconverted, whereas 25/25 (100.0%) of those who received two or three doses seroconverted (**Supplementary Figure 2A**). Notably, 41/41 (100.0%) of the participants in the acute dengue subgroup tested positive for DENV (**Figure 2B**; **Supplementary Figure 2B**).

We examined the DENV serostatus of children administrated Dengvaxia. Among the children that were not ill when studied, 13/59 (22.0%) were seronegative when vaccinated and 46/59 (77.9%) were seropositive; thus, they were immune to DENV when vaccinated. Of the vaccinated children that experienced an acute infection, 10/41 tested negative, that is, there were vaccinated while seronegative. Conversely, 31/41 tested positive. The detection of anti-DENV NS1 IgG could have resulted from a DENV infection prior to vaccination or from the current breakthrough DENV infection.

Based on previous observations that anti-NS1 antibodies from primary flavivirus infections, such as pDENV, pZIKV, or pWNV infection, do not cross-react with flaviviruses from different serocomplexes (32, 41, 42), we reasoned that anti-NS1 IgG’s, induced by breakthrough DENV infection among baseline DENV-naïve Dengvaxia recipients, would appear from days 8 to 12 post-symptom onset similar to pDENV infection (29, 43–46). In contrast, anti-NS1 IgG’s, induced by breakthrough DENV infection among baseline DENV-immune Dengvaxia recipients, would follow the kinetics of sDENV infection, in which antibodies were boosted by breakthrough infection within a few days. As such, we established stringent criteria, in which the presence of anti-DENV NS1 IgG ≤day 7 post-symptom onset was interpreted as baseline DENV immune. The presence of anti-DENV NS1 IgG >day 7 post-symptom onset, however, could be due to previous DENV infection or induced by the current breakthrough infection and therefore was interpreted as undetermined. Of the 31 Dengvaxia recipients with acute dengue, seven serum samples were collected on or before day 7 after the onset of symptoms and tested positive for anti-DENV NS1 IgG, suggesting that these individuals were immune to DENV when vaccinated. Sera were collected from 24 individuals on days 8 to 21 after the onset of dengue symptoms. The immune status of these children at the time of vaccination could not be determined (**Figure 2C**). Of the 23 baseline DENV-naïve Dengvaxia recipients with febrile illnesses studied, 10 had a confirmed acute breakthrough DENV infection, whereas 13 experienced only mild disease. Of the 53 baseline DENV-immune participants that received Dengvaxia and experienced a febrile illness, seven were confirmed as acute dengue and 46 as non-acute dengue (odds ratio=5.05, 95% CI=3.91−6.20) (**Table 2**).

**Table 2:** Dengvaxia recipients: baseline DENV serostatus and disease outcome.

Group^a^	Subgroups^b^ (disease outcome)	DENV serostatus^c^	
		baseline DENV-naïve	baseline DENV-immune
Dengvaxia recipients	Non-acute dengue (n=59)	13	46
	Acute dengue (n=17)	10	7

a Dengvaxia or non-Dengvaxia recipients were determined by history of receiving Dengvaxia.

b DENV RT-PCR test and Panbio dengue IgM-capture ELISA were performed at the RITM. Either one tested positive was designated as acute dengue and both tested negative as non-acute dengue.

c DENV serostatus was determined by the UH DENV1−4 NS1 IgG ELISA; those tested negative were baseline DENV-naïve and those tested positive with sampling time ≤day 7 after symptom onset were baseline DENV-immune. Baseline DENV-naïve Dengvaxia children had a higher risk of symptomatic DENV breakthrough infection than baseline DENV-immune children (odds ratio=5.05, 95% CI=3.91−6.20).

We further examined 17 Dengvaxia recipients with breakthrough DENV infection and found no difference in disease severity between the 10 baseline DENV-naïve (eight diagnosed with dengue with warning signs and two diagnosed with dengue) and seven baseline DENV-immune (five diagnosed with dengue with warning signs, one diagnosed with severe dengue, and one diagnosed with dengue) subgroups, probably due to the small sample size (**Supplementary Table 2**).

### Detection of anti-YFV NS1 IgG among Dengvaxia recipients

**Figure 3:**
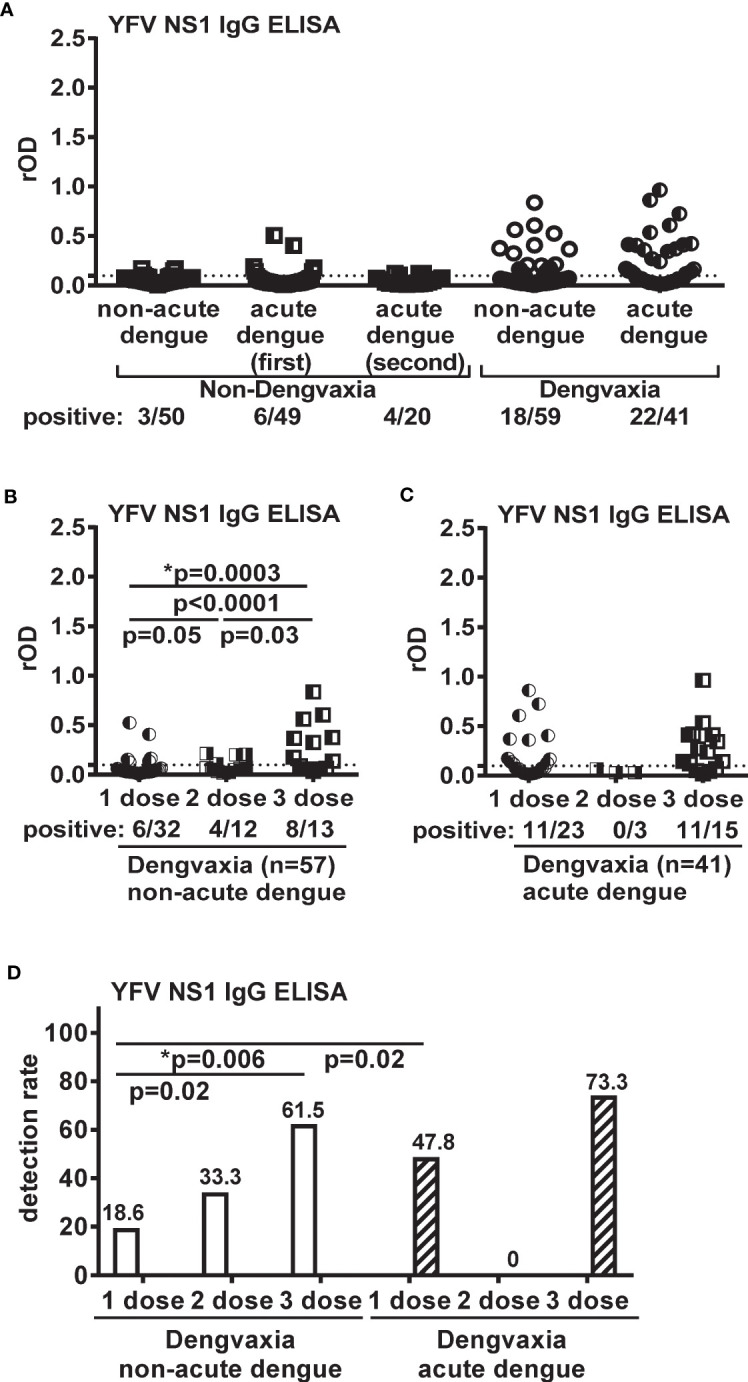
Results of YFV NS1 IgG ELISA for participants from the fever surveillance in the Philippines. **(A)** non-Dengvaxia recipients (non-acute dengue and acute dengue first and second time-point subgroups) and Dengvaxia recipients (non-acute dengue and acute dengue subgroups). **(B, C)** rOD and vaccine dosage for Dengvaxia recipients in non-acute dengue **(B)** and acute dengue **(C)** subgroups. The two-tailed Mann–Whitney test and Kruskal–Wallis test (*) were used to compare rOD between two and three subgroups, respectively. **(D)** Detection rate and vaccine dosage for Dengvaxia recipients in non-acute dengue and acute dengue subgroups. The two-tailed Fisher’s exact test and chi-square test for trend (*) were used to compare detection rate between two and three subgroups, respectively. Dotted lines indicate cutoff rOD. Data represent the mean of two experiments (each in duplicate). rOD, relative OD.

Finally, we explored the possibility of detecting anti-YFV NS1 IgG as a biomarker of Dengvaxia in countries where YFV vaccination or the YFV infection rate is low. As shown in **Figure 3A**, anti-YFV NS1 IgG was detected in 40.0% (40/100) of Dengvaxia recipients. The detection rate was higher among Dengvaxia recipients with acute dengue than in the non-acute dengue subgroup (53.7% [22/41] vs. 30.5% [18/59]; odds ratio=2.64; 95% CI=1.81−3.46), but the difference could not distinguish the two subgroups. Among the 57 Dengvaxia recipients with non-acute dengue that had received a known Dengvaxia dosage, there was an increasing rOD trend of anti-YFV NS1 IgG ELISA (**Figure 3B**) as well as an increasing detection rate (**Figure 3D**) as Dengvaxia increased from one dose to three doses, suggesting a dose–response relationship (p=0.0003 for rOD, Kruskal−Wallis test; p=0.006 for detection rate, chi-square test for trend). For Dengvaxia recipients in the acute dengue subgroup, there was no difference in the rOD or detection rate between the one- and three-dose subgroups (**Figure 3C**).

## Discussion

In this study, we reported that the UH DENV1–4 NS1 IgG ELISA can be used to retrospectively identify the baseline DENV serostatus among Dengvaxia recipients that received one or two doses up to 30 months after vaccination. Moreover, our assay can be employed not only during non-acute dengue but also during breakthrough DENV infection, and has implications for assessing the safety and effectiveness of Dengvaxia in the post-licensure period.

Notably, the DENV NS1 IgG ELISA employed in a previous *post-hoc* Dengvaxia study had a sensitivity and specificity of 95.3% and 68.6%, respectively, based on seven control samples from other flavivirus infections (5, 11, 12). After testing 197 DENV (pDENV and sDENV panels) and 174 non-DENV samples, we showed that the UH DENV1–4 NS1 IgG ELISA had a superior sensitivity/specificity of 96.6%/99.4% (95% CI=93.9−98.0%/98.3−100.0%). Bonaparte et al. evaluated four dengue RDTs and two commercial ELISAs (from Alere and Focus Diagnostics) for pre-vaccination screening and reported favorable specificities (99−100%) for some RDTs and ELISAs, but the varying sensitivities (40−70%) of RDTs were lower than those of ELISAs (≥90%) (16). Similar observations were reported by others including those that used commercial ELISAs from Euroimmun and Abbott (17–19). Recently, Liberal et al. reported an RDT with a sensitivity/specificity of 95.3%/98.0% based on a high-positivity panel (NT_90_ titer≥10), however, the sensitivity dropped to 88.1% for monotypic DENV immune samples (20). Thus, the sensitivity/specificity of our assay is higher than that of RDTs and ELISAs reported for pre-vaccination screening, and higher than that (≥95.0%/98.0%) proposed by dengue experts (14, 15). However, the possibility that anti-DENV NS1 IgG waned over time and was below the limit of detection cannot be ruled out completely. Compared with RDTs, our assay requires a smaller sample volume (<1 µL vs. 5-10 µL for RDTs) and can be performed at lower cost. However, sample processing requires laboratory equipment (ELISA washer and reader vs. point-of-care test) and more time (>2.5 h vs. 20-25 min for RDTs).

**Figure 4:**
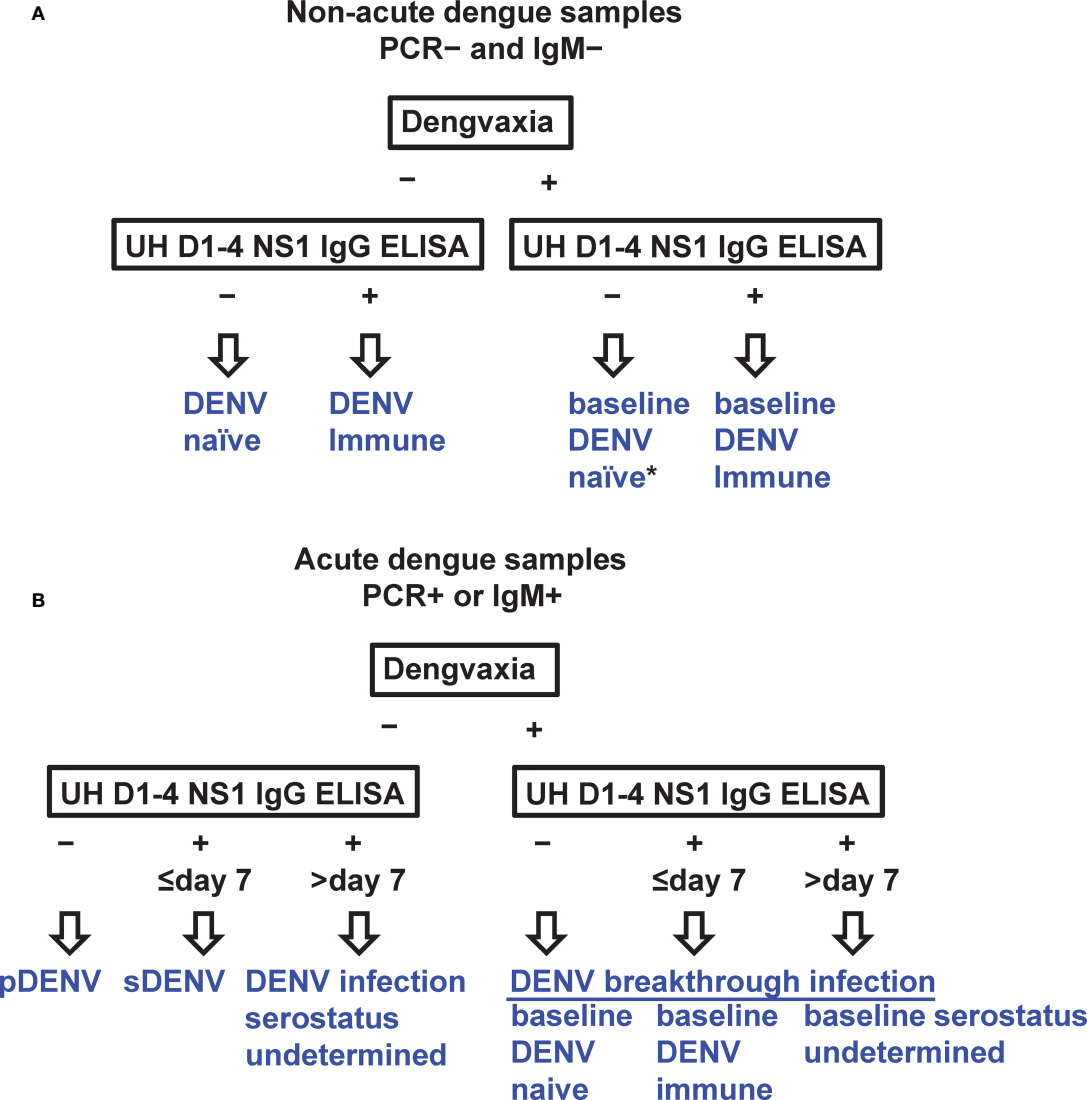
Algorithms using UH DENV1–4 NS1 IgG ELISA to determine baseline DENV serostatus among Dengvaxia recipients as well as non-Dengvaxia recipients. **(A)** Individuals with no evidence of acute dengue (DENV PCR− and IgM−). **(B)** Individuals with acute dengue (DENV PCR+ or IgM+). pDENV, primary DENV infection; sDENV, secondary DENV infection. *An E protein-based IgG ELISA can be used to identify baseline DENV-naïve (anti-DENV NS1 IgG ELISA negative) and non-seroconverted (anti-DENV E IgG ELISA negative) Dengvaxia recipients as a subgroup for further analysis.

Based on the performance of the UH DENV1–4 NS1 IgG ELISA, we proposed an algorithm to determine the baseline DENV serostatus among Dengvaxia recipients with non-acute dengue (**Figure 4A**). A notable proportion of Dengvaxia recipients in the Philippines received only one dose (9, 10); among these individuals, the seroconversion rate (to DENV E protein) was 87.5% (**Supplementary Figure 2A**). An E protein-based IgG ELISA, such as our FL-VLP IgG ELISA, can be used to identify baseline DENV-naïve (anti-DENV NS1 IgG negative) and non-seroconverted (anti-DENV E IgG negative) recipients as a separate subgroup to monitor the outcomes of future DENV infection. Because of the different kinetics of antibody responses during pDENV and sDENV infection, we proposed another algorithm to determine the baseline DENV serostatus among Dengvaxia and non-Dengvaxia recipients with acute dengue (**Figure 4B**). An E protein-based IgG ELISA can also be used to identify baseline DENV-naïve and non-seroconverted recipients as a separate subgroup.

While examining 100 Dengvaxia recipients from the fever surveillance program, we found baseline DENV-naïve children (10/23) had a higher rate of symptomatic breakthrough DENV infection than baseline DENV-immune children (7/53) (odds ratio=5.05, 95% CI=3.91−6.20), demonstrating the feasibility of our assay and algorithms. Of note, a previous report of an increased risk of hospitalization and severe dengue among baseline DENV-seronegative Dengvaxia recipients compared with non-vaccinated controls or baseline DENV-seropositive Dengvaxia recipients was based on clinical outcomes after completion of three doses (12). Our findings provide evidence to support an increased risk of symptomatic breakthrough DENV infection among baseline DENV-naïve Dengvaxia recipients compared to baseline DENV-immune Dengvaxia recipients in real-world settings, where some received only one or two doses of Dengvaxia. Notably, over 1.1 million individuals had received at least one dose of Dengvaxia prior to 2018 when the baseline DENV serostatus was not determined (9). In the Philippines, there were 414,535 dengue cases with 1,546 deaths in 2019, as well as an estimated 5,158 dengue hospitalizations and 1,077 severe dengue cases among Dengvaxia recipients over 4 years (8, 10, 47). As sDENV infection is a risk factor for severe dengue and has been recommended for inclusion in the scoring system to improve triage in the emergency room, information about the baseline DENV-naïve serostatus among Dengvaxia recipients would facilitate clinical assessment and timely treatment when vaccine recipients present with suspected breakthrough DENV infection (22–24). The time interval between the first DENV infection (or exposure) and the second DENV infection, up to 20 years after the first infection, has been shown to affect disease severity (48–50), underscoring the importance of assessing the long-term safety of dengue vaccines through post-licensure studies (8, 9, 26, 27). Based on an estimated DENV seroprevalence of 85% among children aged 9−10 years in the Philippines, 15% of the 880,464 children that received Dengvaxia (132,070) were estimated to be DENV-seronegative. Of the 132,070 DENV-seronegatives, 63,129 would be expected to experience a DENV infection during the 4 years after completion of vaccination based on an estimated 4-year cumulative infection rate of 47.8%; the remaining 68,941 individuals would be Dengvaxia recipients that had not been infected and, if identified, could benefit from improved clinical assessments and timely treatment during breakthrough DENV infection (8, 10, 51). Therefore, the assay and algorithms established in this study have significant public health applications. It can be applied to prospective studies to test Dengvaxia recipients, and identify those classified as baseline DENV seronegative as “at risk” for continuous follow-up (8, 9). The assay can also be applied to fever surveillance program in the Philippines or other dengue-endemic countries to determine the baseline DENV serostatus for Dengvaxia recipients and evaluate disease severity.

The observation that single-dose Dengvaxia recipients had a lower seroconvertion rate (87.5%) to DENV E protein than those receiving two or three doses and that the anti-YFV NS1 IgG-positive rate increased with Dengvaxia dosage suggests that assessing the safety and effectiveness of Dengvaxia should consider its dose effect. This is congruent with a recent report from the Philippines revealing that the effectiveness of single-dose Dengvaxia against hospitalized dengue (26%) and dengue with warning signs (51%) was lower than that of the three-dose, which were reported in vaccine efficacy trials (6, 12, 52).

The overall detection rate of YFV NS1 IgG ELISA was 40% among Dengvaxia recipients, suggesting the difficulty of using anti-YFV NS1 IgG as a biomarker of Dengvaxia in YFV non-endemic countries. Consistent with this, a recent study reported a low detection rate (44,4%) for anti-YFV NS1 IgG among YF-17D vaccinees (53). Similarly, our YFV NS1 IgG ELISA had a detection rate of 31.6% for YF-17D vaccinees (**Supplementary Figure 3**). Collectively, these results suggest that anti-YFV NS1 IgG is not a good serological marker for the YF-17D vaccine as well.

Our study has several limitations. First, the sample size in each group/subgroup was small, and future studies involving larger sample sizes, well-described control panels (including Dengvaxia recipients), and power analysis are needed, especially those aimed at addressing the dose effect of Dengvaxia on the outcomes of breakthrough infection among baseline DENV-naïve versus DENV-immune Dengvaxia recipients. Second, DENV-seropositive status was determined in less than 25% (7/31) of Dengvaxia recipients with acute dengue, likely due to delayed sampling time (>7 days post-symptom onset). Collecting early samples (≤day 7) for Dengvaxia recipients presenting with febrile illness is critical in future studies. Third, a previous study using samples from Dengvaxia vaccine trials reported that the sensitivity and specificity of an E protein-based DENV IgM ELISA to detect breakthrough DENV infection were 93.1% and 77.8%, respectively (54). The reduced specificity, probably due to the cross-reactivity of anti-E antibodies, was unlikely to affect the interpretation of the UH DENV1–4 NS1 IgG ELISA, however, caution should be exercised when using an E protein-based IgM ELISA to detect breakthrough DENV infection among Dengvaxia recipients. Finally, our assay could not distinguish between baseline pDENV and sDENV infections. As individuals with a pDENV infection had a higher risk of severe disease during sDENV infection than those with naïve or sDENV serostatus, new assays that can distinguish the serostatus of pDENV or sDENV infection prior to Dengvaxia vaccination would add to our understanding of the full spectrum of the biological activity of Dengvaxia and the attributes of dengue immunopathogenesis.

## Data availability statement

The original contributions presented in the study are included in the article/**Supplementary Material**. Further inquiries can be directed to the corresponding author.

## Ethics statement

The studies involving human participants were reviewed and approved by the Institutional Review Boards of the University of Hawaii (2022-00201, 2021-00947, CHS #17568), the Research Institute for Tropical Medicine, Philippines (2019-042), and the Kaohsiung Medical University Hospital (KMUH-IRB-960195 and KMUH-IRB-[I]-20170185). The patients/participants provided their written informed consent to participate in this study. Written informed consent to participate in this study was provided by the participants’ legal guardian/next of kin.

## Author contributions

YCD, AS, MJ, and WKW contributed to study design. YCD, AS, MJ, JB, MQ, and MR conducted the experiments. YCD and WKW performed the data analysis. YCD, AS, MJ, and WKW had access to underlying data. AS, MJ, JJT, and WKW contributed to sample collection and funding acquisition. AS and WKW contributed to manuscript writing. All authors contributed to the article and approved the submitted version.

## References

[1] Guzman MG Harris E. Dengue. Lancet (2015) 385:453–65. doi: 10.1016/S0140-6736(14)60572-9 25230594

[2] Bhatt S Gething PW Brady OJ Messina JP Farlow AW Moyes CL. The global distribution and burden of dengue. Nature (2013) 496:504–7. doi: 10.1038/nature12060 PMC3651993 23563266

[3] WHO. Dengue: Guidelines for Diagnosis, Treatment, Prevention and Control. new edition. Geneva, Switzerland (2009). 23762963

[4] Halstead SB Dans LF. Dengue infection and advances in dengue vaccines for children. Lancet Child Adolesc Health (2019) 3:734–41. doi: 10.1016/S2352-4642(19)30205-6 31378686

[5] Wilder-Smith A Hombach J Ferguson N Selgelid M O'Brien K Vannice K. Deliberations of the Strategic Advisory Group of Experts on Immunization on the use of CYD-TDV dengue vaccine. Lancet Infect Dis (2019) 19:e31–8. doi: 10.1016/S1473-3099(18)30494-8 30195995

[6] Hadinegoro SR Arredondo-García JL Capeding MR Deseda C Chotpitayasunondh T Dietze R. Efficacy and long-term safety of a dengue vaccine in regions of endemic disease. N Engl J Med (2015) 373:1195–206. doi: 10.1056/NEJMoa1506223 26214039

[7] Dengue vaccine WHO. WHO position paper – July 2016. Weekly Epidemiological Rec (2016) 91:349–64. 27476189

[8] Halstead SB Katzelnick LC Russell PK Markoff L Aguiar M Dans LR. Ethics of a partially effective dengue vaccine: Lessons from the Philippines. Vaccine (2020) 38:5572–76. doi: 10.1016/j.vaccine.2020.06.079 PMC7347470 32654899

[9] Thomas SJ Yoon IK. A review of Dengvaxia®: development to deployment. Hum Vaccin Immunother (2019) 15:2295–314. doi: 10.1080/21645515.2019.1658503 PMC6816420 31589551

[10] Flasche S Wilder-Smith A Hombach J Smith PG. Estimating the proportion of vaccine-induced hospitalized dengue cases among Dengvaxia vaccinees in the Philippines. Wellcome Open Res (2019) 4:165. doi: 10.12688/wellcomeopenres.15507.1 31815190 PMC6880258

[11] Nascimento EJM George JK Velasco M Bonaparte MI Zheng L DiazGranados CA. Development of an anti-dengue NS1 IgG ELISA to evaluate exposure to dengue virus. J Virol Methods (2018) 257:48–57. doi: 10.1016/j.jviromet.2018.03.007 29567514

[12] Sridhar S Luedtke A Langevin E Zhu M Bonaparte M Machabert T. Effect of dengue serostatus on dengue vaccine safety and efficacy. N Engl J Med (2018) 379:327–40. doi: 10.1056/NEJMoa1800820 29897841

[13] WHO. Dengue vaccine: WHO position paper - September 2018. Weekly Epidemiological Rec (2018) 93:457–76.

[14] Wilder-Smith A Smith PG Luo R Kelly-Cirino C Curry D Larson H. Pre-vaccination screening strategies for the use of the CYD-TDV dengue vaccine: A meeting report. Vaccine (2019) 37:5137–46. doi: 10.1016/j.vaccine.2019.07.016 31377079

[15] Luo R Fongwen N Kelly-Cirino C Harris E Wilder-Smith A Peeling RW. Rapid diagnostic tests for determining dengue serostatus: a systematic review and key informant interviews. Clin Microbiol Infect (2019) 25:659–66. doi: 10.1016/j.cmi.2019.01.002 PMC6543064 30664935

[16] Bonaparte M Zheng L Garg S Guy B Lustig Y Schwartz E. Evaluation of rapid diagnostic tests and conventional enzyme-linked immunosorbent assays to determine prior dengue infection. J Travel Med (2019) 26(8):taz078. doi: 10.1093/jtm/taz078 31616949

[17] Limothai U Tachaboon S Dinhuzen J Hunsawong T Ong-Ajchaowlerd P Thaisomboonsuk B. Dengue pre-vaccination screening test evaluation for the use of dengue vaccine in an endemic area. PloS One (2021) 16:e0257182. doi: 10.1371/journal.pone.0257182 34507347 PMC8432984

[18] Echegaray F Laing P Hernandez S Marquez S Harris A Laing I. Adapting rapid diagnostic tests to detect historical dengue virus infections. Front Immunol (2021) 12:703887. doi: 10.3389/fimmu.2021.703887 34367162 PMC8344047

[19] DiazGranados CA Bonaparte M Wang H Zhu M Lustig Y Schwartz E. Accuracy and efficacy of pre-dengue vaccination screening for previous dengue infection with five commercially available immunoassays: a retrospective analysis of phase 3 efficacy trials. Lancet Infect Dis (2021) 21:529–36. doi: 10.1016/S1473-3099(20)30695-2 PMC9759790 33212068

[20] Liberal V Forrat R Zhang C Pan C Bonaparte M Yin W. Performance evaluation of a dengue IgG rapid diagnostic test designed to determine dengue serostatus as part of prevaccination screening. Microbiol Spectr (2021) 10:e0071121. doi: 10.1128/spectrum.00711-21 PMC9241662 35604130

[21] Daag JV Ylade M Adams C Jadi R Crisostomo MV Alpay R. Evaluation of a new point-of-care test to determine prior dengue infection for potential use in pre-vaccination screening. Clin Microbiol Infect (2021) 27:904–8. doi: 10.1016/j.cmi.2020.08.026 32866651

[22] Sangkaew S Ming D Boonyasiri A Honeyford K Kalayanarooj S Yacoub S. Risk predictors of progression to severe disease during the febrile phase of dengue: a systematic review and meta-analysis. Lancet Infect Dis (2021) 21:1014–26. doi: 10.1016/S1473-3099(20)30601-0 PMC8240557 33640077

[23] Htun TP Xiong Z Pang J. Clinical signs and symptoms associated with WHO severe dengue classification: a systematic review and meta-analysis. Emerg Microbes Infect (2021) 10:1116–28. doi: 10.1080/22221751.2021.1935327 PMC8205005 34036893

[24] Yuan K Chen Y Zhong M Lin Y Liu L. Risk and predictive factors for severe dengue infection: A systematic review and meta-analysis. PloS One (2022) 17:e0267186. doi: 10.1371/journal.pone.0267186 35427400 PMC9012395

[25] Arien KK Wilder-Smith A. Dengue vaccine: reliably determining previous exposure. Lancet Global Health (2018) 6:e830–1. doi: 10.1016/S2214-109X(18)30295-X 29941282

[26] WHO. Guidelines on the quality, safety and efficacy of dengue tetravalent vaccines (live, attenuated). (Geneva, Switzerland: WHO) (2011).

[27] Wichmann O Vannice K Asturias EJ de Albuquerque Luna EJ Longini I Lopez AL. Live-attenuated tetravalent dengue vaccines: The needs and challenges of post-licensure evaluation of vaccine safety and effectiveness. Vaccine (2017) 35:5535–42. doi: 10.1016/j.vaccine.2017.08.066 28893477

[28] Pierson TC Diamond MS Flaviviruses. Knipe DM Howley PM. Fields virology. 6th ed. (Philadelphia: Lippincott William & Wilkins) (2013) p. 747–94.

[29] Lai CY Tsai WY Lin SR Kao CL Hu SP King CC. Antibodies to envelope glycoprotein of dengue virus during the natural course of infection are predominantly cross-reactive and recognize epitopes containing highly conserved residues at the fusion loop of domain II. J Virol (2008) 82:6631–43. doi: 10.1128/JVI.00316-08 PMC2447043 18448542

[30] Lanciotti RS Kosoy OL Laven JJ Velez JO Lambert AJ Johnson AJ. Genetic and serologic properties of Zika virus associated with an epidemic, Yap State, Micronesia, 2007. Emerg Infect Dis (2008) 14:1232–9. doi: 10.3201/eid1408.080287 PMC2600394 18680646

[31] Guidance for US. Laboratories Testing for Zika Virus Infection. (Atlanta, GA, USA: CDC’s). (2019). Available at: http://www.cdc.gov/zika/laboratories/lab-guidance.html.

[32] Tyson J Tsai WY Tsai JJ Brites C Mässgård L Youn HH. Combination of non-structural protein 1-based enzyme-linked immunosorbent assays can detect and distinguish various dengue virus and Zika virus infections. J Clin Microbiol (2019) 57:e01464–18. doi: 10.1128/JCM.01464-18 PMC6355536 30429254

[33] Tsai WY Driesse K Tsai JJ Granat RS Jenkins O Hsieh SC. Enzyme-linked immunosorbent assays using virus-like particles containing mutations of conserved residues on envelop protein can distinguish three flavivirus infections. Emerg Microbe Infect (2020) 9:1722–32. doi: 10.1080/22221751.2020.1797540 PMC7473235 32684139

[34] Tsai JJ Liu CK Tsai WY Liu LT Tyson J Tsai CY. Seroprevalence of dengue in two districts of Kaohsiung city after the largest dengue outbreak in Taiwan since world war II. PloS Negl Trop Dis (2018) 12:e0006879. doi: 10.1371/journal.pntd.0006879 30356316 PMC6218099

[35] Tsai WY Chen HL Tsai JJ Dejnirattisai W Jumnainsong A Mongkolsapaya J. Potent neutralizing human monoclonal antibodies preferentially target mature dengue virus particles: implication for novel strategy of dengue vaccine. J Virol (2018) 92:e00556–18. doi: 10.1128/JVI.00556-18 PMC6232466 30185598

[36] Wang WK Chen HL Yang CF Hsieh SC Juan CC Chang SM. Slower rates of clearance of viral load and virus-containing immune complexes in patients with dengue hemorrhagic fever. Clin Infect Dis (2006) 243:1023–30. doi: 10.1086/507635 16983615

[37] Herrera BB Tsai WY Brites C Luz E Pedroso C Drexler JF. T cell responses to nonstructural protein 3 distinguish infections by dengue and Zika viruses. mBio (2018) 9:e00755–18. doi: 10.1128/mBio.00755-18 PMC6083909 30087165

[38] Johnson BW Russell BJ Lanciotti RS. Serotype-specific detection of dengue viruses in a fourplex realtime reverse transcriptase PCR assay. J Clin Microbiol (2005) 43:4977–83. doi: 10.1128/JCM.43.10.4977-4983.2005 PMC1248506 16207951

[39] Biggs JR Sy AK Ashall J Santoso MS Brady OJ Reyes MAJ. Combining rapid diagnostic tests to estimate primary and post-primary dengue immune status at the point of care. PloS Negl Trop Dis (2022) 16:e0010365. doi: 10.1371/journal.pntd.0010365 35507552 PMC9067681

[40] Frey A Di Canzio J Zurakowski D. A statistically defined endpoint titer determination method for immunoassays. J Immunol Methods (1998) 221:35–41. doi: 10.1016/S0022-1759(98)00170-7 9894896

[41] Balmaseda A Stettler K Carrera RM Collado D Jin X Zambrana JV. A novel antibody-based assay discriminates Zika virus infection from other flaviviruses. Proc Natl Acad Sci USA (2017) 114:8384–9. doi: 10.1073/pnas.1704984114 PMC5547631 28716913

[42] Balmaseda A Zambrana JV Collado D García N Saborío S Elizondo D. Comparison of four serological methods and two reverse transcription-PCR assays for diagnosis and surveillance of Zika virus infection. J Clin Microbiol (2018) 56:e01785–17. doi: 10.1128/JCM.01785-17 PMC5824050 29305550

[43] Gubler DJ. Dengue and dengue hemorrhagic fever. Clin Microbiol Rev (1998) 11:480–96. doi: 10.1128/CMR.11.3.480 PMC88892 9665979

[44] Busch MP Kleinman SH Tobler LH Kamel HT Norris PJ Walsh I. Virus and antibody dynamics in acute West Nile virus infection. J Infect Dis (2008) 198:984–93. doi: 10.1086/591467 18729783

[45] Shu PY Chen LK Chang SF Yueh YY Chow L Chien LJ. Comparison of capture immunoglobulin M (IgM) and IgG enzyme-linked immunosorbent assay (ELISA) and nonstructural protein NS1 serotype-specific IgG ELISA for differentiation of primary and secondary dengue virus infections. Clin Diagn Lab Immunol (2003) 10:622–30. doi: 10.1128/CDLI.10.4.622-630.2003 PMC164246 12853395

[46] Shu PY Chang SF Yueh YY Chow L Chien LJ Kuo YC. Current status of dengue diagnosis at the Center for Disease Control, Taiwan. Dengue Bull (2004) 28:107–17.

[47] WHO. Regional Office for the Western Pacific. Dengue situation update, Dengue in Philippines. West Pacific, Manila (2019). Available at: https://apps.who.int/iris/handle/10665/279856.

[48] Guzman MG Kouri G Valdes L Bravo J Vazquez S Halstead SB. Enhanced severity of secondary dengue 2 infections occurring at an interval of 20 compared with 4 years after dengue 1 infection. PAHO J Epidemiol (2002) 81:223–7. 10.1590/s1020-49892002000400003 12049030

[49] Montoya M Gresh L Mercado JC Williams KL Vargas MJ Gutierrez G. Symptomatic versus inapparent outcome in repeat dengue virus infections is influenced by the time interval between infections and study year. PloS Negl Trop Dis (2013) 7:e2357. doi: 10.1371/journal.pntd.0002357 23951377 PMC3738476

[50] Anderson KB Gibbons RV Cummings DA Nisalak A Green S Libraty DH. A shorter time interval between first and second dengue infections is associated with protection from clinical illness in a school-based cohort in Thailand. J Infect Dis (2014) 209:360–8. doi: 10.1093/infdis/jit436 PMC3883164 23964110

[51] Coudeville L Baurin N Vergu E. Estimation of parameters related to vaccine efficacy and dengue transmission from two large phase III studies. Vaccine (2016) 34:6417–25. doi: 10.1016/j.vaccine.2015.11.023 26614588

[52] Ylade M Agrupis KA Daag JV Crisostomo MV Tabuco MO Sy AK. Effectiveness of a single-dose mass dengue vaccination in Cebu, Philippines: A case-control study. Vaccine (2021) 39:5318–25. doi: 10.1016/j.vaccine.2021.07.042 34373121

[53] Raulino R Thaurignac G Butel C Villabona-Arenas CJ Foe T Loul S. Multiplex detection of antibodies to Chikungunya, O'nyong-nyong, Zika, Dengue, West Nile and Usutu viruses in diverse non-human primate species from Cameroon and the Democratic Republic of Congo. PloS Negl Trop Dis (2021) 15:e0009028. doi: 10.1371/journal.pntd.0009028 33476338 PMC7853492

[54] Plennevaux E Moureau A Arredondo-García JL Villar L Pitisuttithum P Tran NH. Impact of dengue vaccination on serological diagnosis: insights from phase III dengue vaccine efficacy trials. Clin Infect Dis (2018) 66:1164–72. doi: 10.1093/cid/cix966 PMC5888923 29300876

