# Supplementary material for: Identification of prior dengue-naïve Dengvaxia recipients with an increased risk for symptomatic dengue during fever surveillance in the Philippines
Source: Front Immunol. 2023 Jul 24;14:1202055. doi: 10.3389/fimmu.2023.1202055 (PMC10405517; doi:10.3389/fimmu.2023.1202055)
Supplement: Supplementary file 1 [file DataSheet_1.pdf]

Supplementary Table 1 Numbers, sources and basic information of control serum/plasma panels

Panel	No. of samples /subjects	Sources (No. of samples /subjects)	Sampling time (post-symptom onset)	Country and year (reference)	Confirmation methods
pDENV	56/40	seroprevalence study (12/12)	6M−31Y	Taiwan, 2015−16 (34)	Neutralization test[a]
		DENV study (11/11)	1M−5.6Y	Taiwan, 2001−9 (32)	RT-PCR
		DENV study (11/11)	2.7M−10M	Hawaii, 2015 (32)	RT-PCR
		DENV study (22/6)	1M−1.5Y	Nicaragua, 2015 (32)	RT-PCR
sDENV	113/113	seroprevalence study (24/24)	1M−30Y	Taiwan, 2015−16 (34)	Neutralization test[a]
		DENV study (57/57)	<1M−6Y	Taiwan, 2001−9 (32)	RT-PCR
		DENV study (2/2)	3M−5.6M	Hawaii, 2015 (32)	RT-PCR
		DENV study (30/30)	3M−1Y	Nicaragua, 2015 (32)	RT-PCR
DENV-negative	140/140	seroprevalence study (140/140)	NA	Taiwan, 2015−16 (34)	Neutralization test[a] or multiple ELISAs
pZIKV	13/13	ZIKV study (13/13)	1.5M−1.3Y	Brazil, 2016−17 (37)	Neutralization test[a]
ZIKVwprDENV	28/22	ZIKV study (28/22)	3M−2.3Y	Brazil, 2016−17 (37)	Neutralization test[a]
pWNV	18/18	WNV study (18/18)	<1M	U.S. 2006−15 (32)	TMA[b]
JEV	3/3	JEV cases (3/3)	<1M	Taiwan, 2013−16	RT-PCR

[a]Microneutralization test was described previously (35,37).
[b]Index samples tested positive for WNV transcription-mediated amplification (TMA), IgM and IgG from blood donors at the American Red Cross (32).
Abbreviation: pDENV, primary DENV infection; sDENV, secondary DENV infection; pWNV, primary WNV infection; pZIKV, primary ZIKV infection; ZIKVwprDENV, ZIKV infection with previous DENV infection; JEV, JEV infection; NA, not applicable.

Supplementary Table 2 Basic and clinical information of Dengvaxia recipients with acute dengue

ID (n=41)	Age[a]	Sex	Dengvaxia dose	Sampling day[b]	PCR[c]	Dengue IgM[c]	Fever	Clinical Diagnosis[d]	UH DENV1-4 NS1 IgG ELISA[e]	Baseline DENV serostatus[f]
DE18-0071	9	F	1	7	−	+	Yes	W	−	naïve
DE18-10052	13	M	2	9	−	+	Yes	W	−	naïve
DE18-10921	11	F	1	9	−	+	Yes	N	−	naïve
DE18-12321	13	M	1	9	−	+	Yes	W	−	naïve
DE18-13571	11	M	1	7	−	+	Yes	W	−	naïve
DE18-13575	11	M	3	11	−	+	Yes	W	−	naïve
DE18-13650	11	F	1	11	−	+	Yes	W	−	naïve
DE18-1473	12	F	3	9	−	+	Yes	W	−	naïve
DE18-14046	12	F	2	7	−	+	Yes	N	−	naïve
DE18-14085	15	F	1	8	−	+	Yes	W	−	naïve
DE18-0907	11	M	1	7	−	+	Yes	W	+	immune
DE18-10562	12	F	3	7	−	+	Yes	N	+	immune
DE18-11691	11	F	1	7	−	+	Yes	W	+	immune
DE18-12794	10	F	1	7	−	+	Yes	W	+	immune
DE18-13573	15	M	1	7	−	+	Yes	W	+	immune
DE18-7245	11	F	3	7	−	+	Yes	S	+	immune
DE18-13852	15	F	1	7	−	+	Yes	W	+	immune
DE18-0926	12	F	3	8	−	+	Yes	N	+	UD
DE18-10062	12	F	3	8	−	+	Yes	W	+	UD
DE18-10091	11	F	1	8	−	+	Yes	W	+	UD
DE18-10256	12	F	3	16	−	+	Yes	W	+	UD
DE18-10420	12	F	3	10	−	+	Yes	W	+	UD
DE18-1090	16	M	1	9	−	+	Yes	W	+	UD
DE18-11662	12	M	3	8	−	+	Yes	W	+	UD
DE18-11860	17	M	1	8	−	+	Yes	W	+	UD
DE18-12656	12	M	1	9	−	+	Yes	N	+	UD
DE18-12856	15	M	1	8	−	+	Yes	W	+	UD
DE18-13064	15	M	1	9	−	+	Yes	W	+	UD
DE18-13390	10	M	1	9	−	+	Yes	N	+	UD
DE18-13652	12	F	1	8	−	+	Yes	W	+	UD
DE18-13746	7	F	1	21	−	+	Yes	N	+	UD
DE18-1687	12	M	3	10	−	+	Yes	W	+	UD
DE18-2488	12	M	1	9	−	+	Yes	N	+	UD
DE18-3322	12	M	1	12	−	+	Yes	N	+	UD
DE18-3760	11	F	3	14	−	+	Yes	NA	+	UD
DE18-5627	11	M	3	11	−	+	Yes	N	+	UD
DE18-5994	11	M	3	8	−	+	Yes	W	+	UD
DE18-6462	12	M	3	16	−	+	Yes	NA	+	UD
DE18-7062	11	F	3	11	−	+	Yes	NA	+	UD
DE18-8064	11	M	1	20	−	+	Yes	W	+	UD
DE18-13858	13	F	2	9	−	+	Yes	W	+	UD

[a]Age at the time of receiving Dengvaxia.
[b]Sampling day after symptom onset. The day of symptom (fever) onset is designated as day 1.
[c]DENV RT-PCR test and Panbio dengue IgM-capture ELISA were performed at the RITM. Either one tested positive was acute dengue and both tested negative were non-acute dengue.
[d]Clinical diagnosis was based on WHO case classification (3). N, dengue; W, dengue with warming signs; S, severe dengue; NA, not available.
[e]UH DENV1–4 NS1 IgG ELISA was described in the Materials and Methods.
[f]Baseline DENV serostatus was determined based on the algorithms described in Figure 4. UD, undetermined.

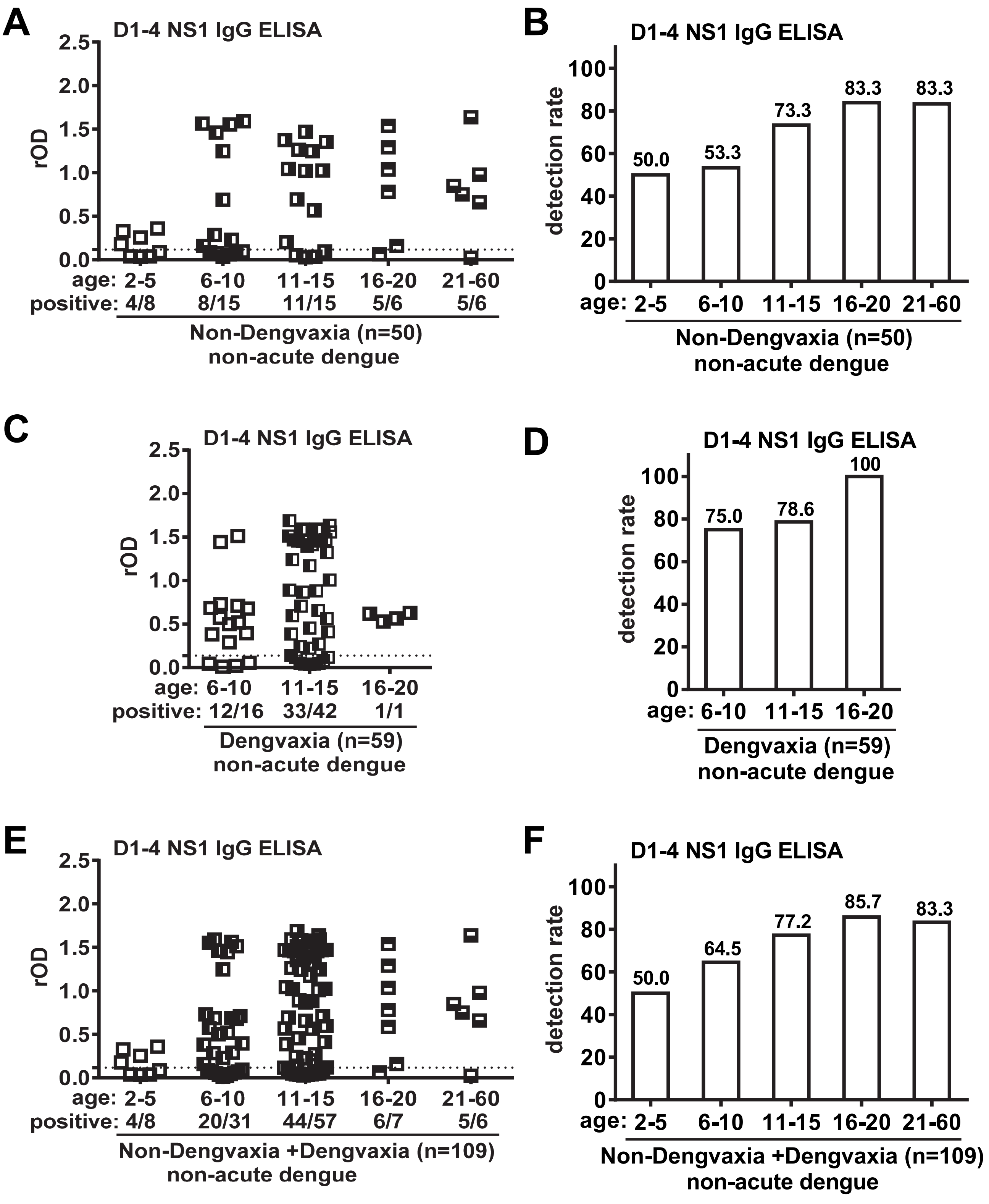

Supplementary Figure 1

Age-specific seroprevalence of dengue based on UH DENV1−4 NS1 IgG in study participants without evidence of acute dengue. (A−F) rOD and detection rate for participants with non-acute dengue including non-Dengvaxia recipients (A,B), Dengvaxia recipients (C,D), and combined non-Dengvaxia and Dengvaxia recipients together (E,F). Dotted lines indicate cutoff rOD. Data represent the mean of two experiments (in duplicate). rOD, relative OD.

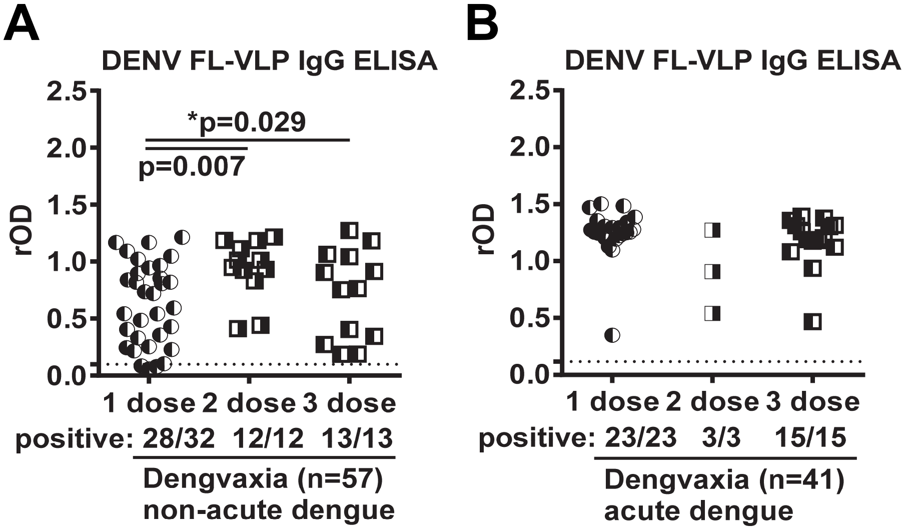

Supplementary Figure 2

Anti-DENV E seroconversion based on DENV FL-VLP IgG ELISA and vaccine dosage for Dengvaxia recipients. (A,B) rOD, seroconversion (positive) rate, and vaccine dosage for Dengvaxia recipients in non-acute dengue (A) and acute dengue (B) subgroups. The two-tailed Mann−Whitney test and Kruskall−Wallis test (*) were used to compare rOD between two and three subgroups, respectively. rOD, relative OD.

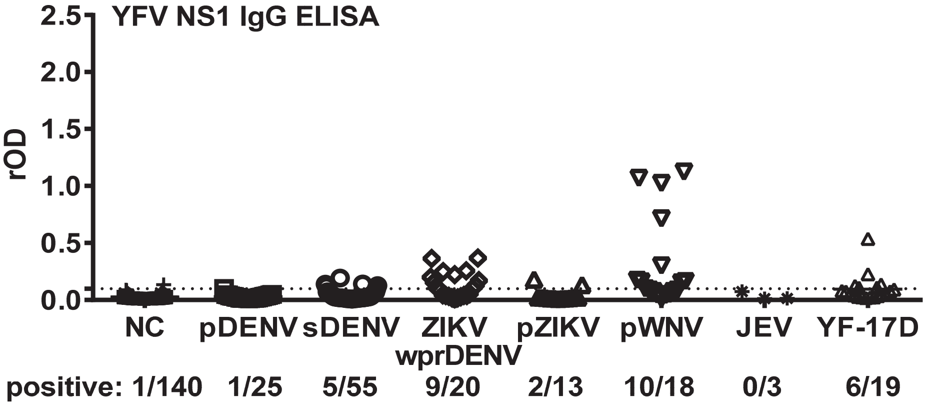

Supplementary Figure 3

The performance of YFV NS1 IgG ELISA using different panels of samples. rOD and detection rates in different panels including YF-17D vaccinees, DENV-negative, pDENV, sDENV, ZIKVwprDENV, pZIKV, pWNV, and JEV panels. Sensitivity/specificity=31.6%/89.8% for YF-17D vaccinees. The history of YF-17D vaccine was not known for ZIKVwprDENV, pZIKV, and pWNV panels. Dotted lines indicate cutoff rOD. Data represent the mean of two experiments (in duplicate) for most panels except one experiment (in duplicate) for ZIKVwprDENV, pZIKV, and pWNV panels. rOD, relative OD; NC, negative control of DENV-negative samples; pDENV, primary DENV infection; sDENV, secondary DENV infection; pWNV, primary WNV infection; pZIKV, primary ZIKV infection; ZIKVwprDENV, ZIKV infection with previous DENV infection; JEV, JEV infection.
